# Global survey of rice breeders to investigate characteristics and willingness to adopt alternative breeding methods

**DOI:** 10.1186/s40066-018-0191-3

**Published:** 2018-06-25

**Authors:** Bert Lenaerts, Bertrand C. Y. Collard, Matty Demont

**Affiliations:** 1 Centre for Environmental Sciences, UHasselt, Hasselt, Belgium; 2 Agri-food Policy Platform, International Rice Research Institute (IRRI), Los Baños, Philippines; 3 Department of Earth and Environmental Sciences, KU Leuven, Leuven, Belgium; 4 Plant Breeding Platform, International Rice Research Institute (IRRI), Los Baños, Philippines

**Keywords:** Rice breeding, Pedigree method, Rapid generation advance, Breeding cycle, Gender, Technology adoption

## Abstract

**Background:**

Despite the critical role rice breeders play to ensure food security, there is a lack of information regarding their current socio-economic characteristics, constraints and attitudes towards technology adoption. Some key concepts like budget, experience, local ecosystems, level of education and even main breeding method have hardly been surveyed in the past. This not only clouds any policy making regarding scientists in national agricultural research programmes, it also makes it difficult to assess the needs and problems local rice breeders face around the world.

**Methods:**

A global online survey was conducted reaching 189 rice breeders from 51 rice-growing countries around the world. The questionnaire was structured according to an adoption framework we proposed from the literature. We specifically investigated their attitudes to adopting an alternative breeding method called rapid generation advance (RGA) (also known as single seed descent). To provide some historical perspective, we compare our results with those reported by Hargrove (Rice breeders in Asia: a ten-country survey of their backgrounds, attitudes, and use of genetic materials, 1978), the only published survey on rice breeders.

**Results:**

Overall, rice breeders are highly educated and have a long experience with their main breeding method. However, a gender gap with respect to education seems to persist. Large variation in resources (staff, land and budget) was observed with a small number of resource-rich institutes and a large number of resource-poor institutes. Most rice breeders are focused on breeding for irrigated conditions. Most breeders have a relatively high degree of risk taking and time preference towards shorter breeding cycles. The majority of breeders are aware of RGA and its benefits with more than half having observed RGA in practice. Finally, breeders are confident in the RGA technique and estimate its resource savings to be substantial.

**Conclusions:**

Breeders’ willingness to adopt RGA was remarkably high. Surprisingly, adoption of RGA remains low (4% as main method). This may suggest that the benefits of using the RGA method still need to be further demonstrated in rice breeding. Our results could be useful to develop targeted extension material or interventions for implementing new technologies, which could be useful to high-level agricultural managers, international research centres and aid agencies.

## Background

In the past 50 years, rice farming has already undergone a great transformation, of which a large part can be attributed to improved varieties. The first rice semi-dwarf varieties were developed and released in the 1960s and 1970s and had a dramatic impact on production [6, 14]. However, rice breeding is a slow and tedious process, although new technologies have cut the time needed to develop and test new varieties. Recently two challenges are appearing on the horizon. The first, and probably most daunting challenge, is the increasing demand for rice that will put pressure on rice breeding; rice production will need to increase to feed the global population in future [31]. There are many anticipated challenges to increase rice production in the twenty-first century, including decreasing land area devoted to agriculture, increased environmental pollution, and land degradation [5, 20]. Climate change is also likely to adversely affect yield in the future [27]. This poses a dangerous mix of mutually reinforcing factors that will lead to a strong increase in demand. This increase will be especially strong in the developing world where not only poverty and population growth are high but also climate change is expected to hit hardest [22]. These factors will not only increase the general level of demand but accelerate the speed of increase as well. This means breeding methods need not only to deliver better rice varieties, but deliver them more quickly as well. These challenges require a drastic rethinking of current breeding methods to be able to meet future demands [1, 11, 12].

In the past, International Agricultural Research Centres (IARCs), such as the International Rice Research Institute (IRRI), developed and disseminated a range of varieties and technologies needed by the highly diverse rice-growing world. However, during the last 10–20 years, the continuous development of new varieties and improvement of farming methods has been undertaken by National Agricultural Research and Extension Systems (NARES). Reduced funding to IARCs and an increasing reliance on local knowledge and expertise have made NARES partners indispensable in the development of new rice varieties. This trend is visible in the increased operational capability of many NARES partners [4] and IARCs’ gradual shift from delivering finished varieties to providing only germplasm [14]. Unfortunately, despite a long and fruitful partnership with NARES breeders, little work has been reported about their characteristics. Some key concepts like budget, experience, local ecosystems, level of education and even main breeding method have hardly been surveyed in the past. This not only clouds any policy making regarding NARES scientists, it also makes it difficult to assess the needs and problems local rice breeders face around the world.

Despite the vital importance of rice breeding, there is almost a complete lack of information regarding socioeconomic status or current constraints of rice breeders and their currently used breeding methods. While many policy makers emphasise the need for new and innovating breeding methods, those same policy makers are left in the dark about the current status of breeding technology in the field. Following this, questions about, for example, institutional resources or educational background of rice breeders have long remained unanswered. Some key breeder characteristics were surveyed in the past by Hargrove [17], the only previous survey conducted on rice breeders. This study suffers some limitations as it only surveyed a small number of Asian breeders on a limited set of characteristics. The author himself points to the importance of agricultural scientists and the fact that little is known about them. Unfortunately, his study received little follow-up research.

Due to the anticipated future challenges of increasing rice production, a superior rice breeding method called rapid generation advance (RGA) was recently proposed for routine adoption for breeding of inbred rice varieties [10]. Rapid generation advance (RGA) has been used in many other plant breeding programmes for field crops [28] and has been described in many textbooks. Briefly, the method is used to speed up the process of line fixation (i.e. making lines genetically homozygous) in self-pollinated crops. Usually, this involves high-density planting of breeding populations (usually in a greenhouse) which causes plants to flower and set seed earlier compared to the field, and hence generations can be advanced more quickly. The main advantages of using RGA include quicker plant breeding, decreased costs for breeding programmes and institutes, and increased public benefits because new varieties may be available earlier than using other methods [23]. In essence, the RGA method is technically simple and requires considerably less resources compared to other rice breeding methods [10]. The cost reduction arises from: (a) significantly lower space (i.e. field space) requirements per generation; (b) drastically lower labour demand because no screening is required; and (c) saving materials and tools for data collection [24]. When combined, this could lead to a large decrease in breeding costs. Secondly, public benefits increase because varieties can be released earlier [26]—reducing “time to market”—and because—according to selection theory—RGA will increase the rate of genetic gain or rate of yield increase over time. Thirdly, labour can be more controlled over the year and the RGA method is highly flexible, which means it can be easily adapted to different, existing breeding programmes. Despite the successful implementation of RGA in other crops and a small number of empirical studies confirming its effectiveness, its use for rice breeding has been extremely limited [15, 18, 24]. RGA was widely used at various times at IRRI and was recently adopted on a large scale for irrigated rice breeding at IRRI in order to increase rice breeding efficiency [10].

To fill the current knowledge gap about socio-economic factors about breeders, their methods and attitudes, we investigated the current status of rice breeding programmes and provide data about the global population of rice breeders using an extensive list of characteristics. To achieve this, we contextualised an adoption framework from the literature [13] to the case of plant breeding and conducted a global online survey of rice breeders to present some insights into rice breeders and their methods. We also specifically investigated their attitudes to adopting RGA instead of their current method. To provide some historical perspective, we compare our results with those reported by Hargrove [17], the only published survey on rice breeders.

## Methods

### Adoption framework

Over the years a variety of technology adoption frameworks has been developed in the context of farmers [29, 32]. More recently, attention has focused on the concept of willingness to adopt (WTA). In contrast to conventional adoption studies, studies on willingness to adopt look from an ex ante point of view (i.e. before adoption takes place) at the adoption of technology by farmers, or more accurately, to their stated willingness to adopt a certain technology. Farmers are given a scenario explaining all relevant characteristics of the new technology after which they are asked whether they are willing to adopt. In other words, their willingness to adopt is a stated, hypothetical decision conditional on the scenario given. In most cases, this stated willingness to adopt is seen as a binary (yes/no) choice [2, 8, 16, 25], but it can also be represented by a categorical or continuous variable, like a technology package [3] or the area to be cultivated with the new technology [7]. A willingness to adopt approach is especially useful when adoption is low.

Nonetheless, farm structures are not comparable to the large organisational structures in which rice breeding programmes are embedded. Unless they are operating under contract farming, farmers have full decision-making power over input use and production technology as they both manage and own the farm. Breeders in a breeding station, in contrast, often report to a breeding department head or head of the institute. Also, farms are either small-scale—often producing for subsistence—or businesses with a private profit-maximising goal. Institutes, on the other hand, operate on a larger scale and want to maximise profits (private sector) or social welfare (public sector). These organisational differences are most likely to influence the adoption process. Nevertheless, to date, no adoption studies exist that look specifically at the adoption process of new technologies within agricultural organisations, like breeding institutes or programmes.

**Fig. 1:**
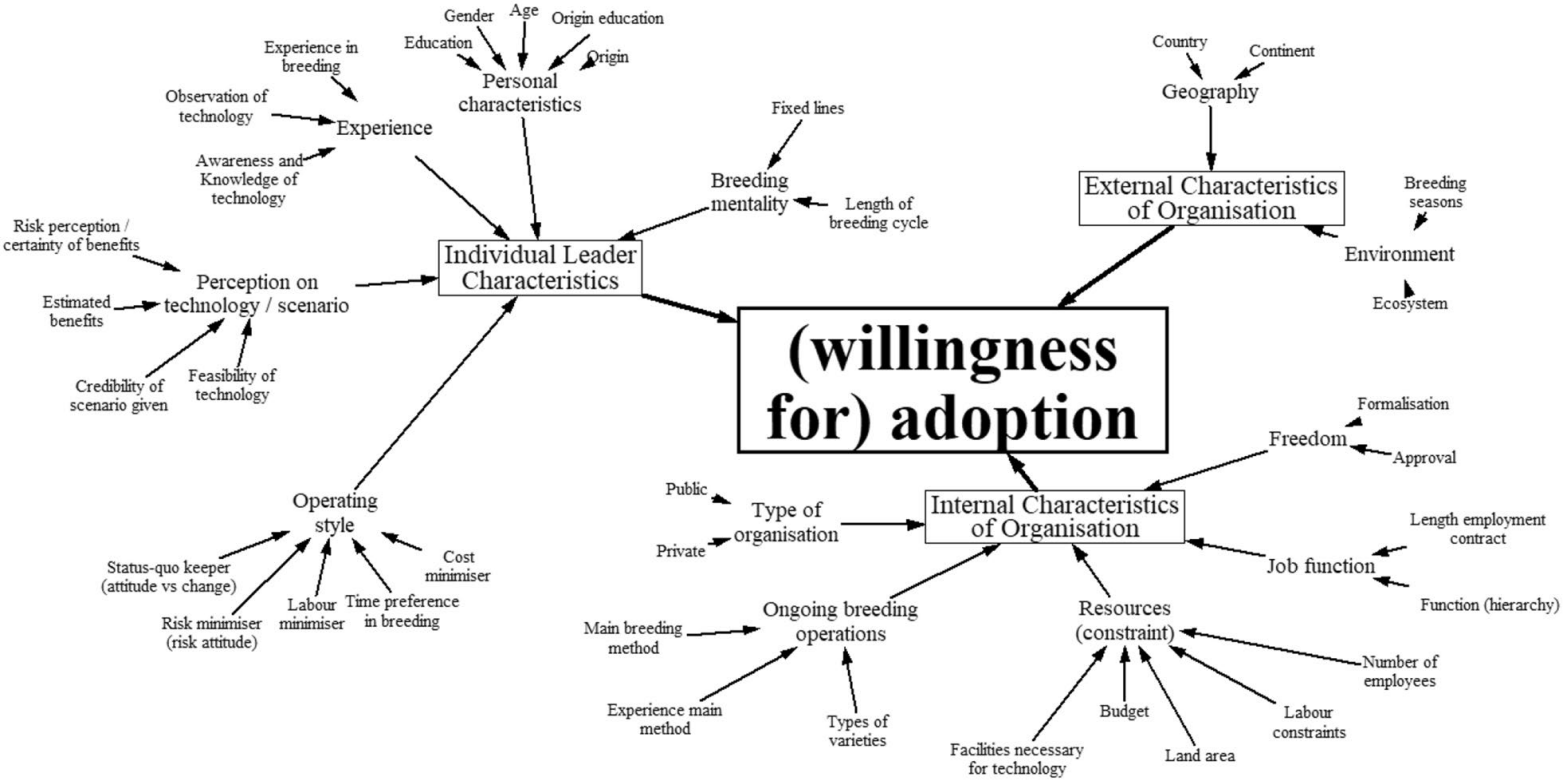
Framework for the adoption of new technologies in plant breeding *Source*: England et al. [13], Rogers [30], Hall et al. [16], Mathijs [25], Blazy et al. [2], and expert elicitation from the International Rice Research Institute

To understand the adoption of a technology within an organisation, we followed guidelines from England et al. [13], who developed a framework for the adoption of information technology by health (care) organisations, which in turn was based on Rogers’ [30] seminal work on adoption and diffusion of innovations. The authors apply innovation diffusion theory to identify variables that either influence the rate of adoption by organisations (an “organisation’s readiness to adopt”) or the rate at which a technology diffuses (the “technology’s readiness to be adopted”). For this article, we are mainly interested in the former. According to England et al. [13], the three categories that influence an “organisation’s readiness to adopt” are: (a) individual leader characteristics; (b) internal characteristics of the organisation, and (c) external characteristics of the organisation. In Fig. 1, we contextualise this framework to the case of plant breeding to study plant breeders’ willingness to adopt new technologies.

Individual leader characteristics are influenced by personal characteristics, experience, perception of the technology, breeding mentality and operating style. Personal characteristics include age, gender and education while experience is understood as breeding experience and awareness about and/or observation of specific breeding technologies in the field. Perception can be measured as the credibility breeders attach to the scenario offered to them. Breeding mentality is captured in the number of generations it takes to fix a line and the number of years before yield trials can start. Operating style looks at breeders’ preferences regarding cost, labour, time, risk and their innovativeness.

Internal characteristics of the organisation comprise freedom, resources, the ongoing breeding operations, the type of organisation and the job function. Freedom is determined by the possibility to implement and experiment with new techniques—called the “level of formalisation” by England et al. [13]. Resources are understood as the sum of land, budget, labour—both staff and seasonal—and specific facilities such as a greenhouse. Constraints to or abundance of one of these may influence a breeder’s decision to adopt a specific breeding technology since breeding methods can differ substantially in resource use. Furthermore, factors related to their job as breeders might play a role, such as the length of their employment contract and their position in the institute’s hierarchy. Given the duration of a standard breeding programme, having a permanent job employment contract might encourage breeders to take long-term decisions like adopting a new breeding method. Lastly, the use of inbreds and/or hybrids along with the main breeding method and the public or private nature of the institute are factors of importance.

Finally, the external characteristics of the organisation are captured through the number of breeding seasons a year that can be conducted, the ecosystem and the geographic location of the institute.

### Survey

A global online survey using Google Forms was conducted reaching 189 rice breeders from 51 rice-growing countries around the world. Our target population consisted of all types of rice breeders working at IARCs (excluding current IRRI breeders) or NARES institutes around the world. Only currently employed rice breeders were considered (i.e. recently retired breeders were excluded). Some rice breeders who were not actively involved in breeding line development (e.g. only involved in testing advanced lines obtained from elsewhere), and people considered to be molecular breeders or molecular geneticists were also excluded from the survey, because they were not routinely involved in core breeding operations. Our focus was on breeders worldwide although the interest was more on breeders from Asia, Africa and Latin America. Due to unavailability of Google services in China, very few Chinese breeders responded (by completing the survey as an email attachment).

Email addresses of rice breeders in the world were compiled from IRRI’s global rice breeding network. Most current IRRI breeders, scientists, country office representatives and key members in the global rice breeding community were consulted to provide nominations. IRRI has an extensive global rice breeding network from ongoing programmes (e.g. International Network for the Genetic Evaluation of Rice, INGER and Temperate Rice Research Consortium, TRRC) or currently funded research programmes (e.g. stress tolerant rice for Africa and South Asia, STRASA). Hybrid breeders were also included using contacts of the IRRI-led Hybrid Rice Development Consortium (HRDC). Breeders from the Africa Rice Center (AfricaRice) and the International Center for Tropical Agriculture (CIAT) were contacted to provide nominations for Africa and Latin America, respectively. Recent participants of the annual IRRI rice breeding course (2011–2013) and visitors who were rice breeders were also included. Note that no sampling frame was used to ensure representativeness of the sample due to the limitation of not being able to reach all breeders.

An initial email was sent to 415 putative rice breeders from each country requesting them to nominate rice breeders that report to them, or who were colleagues. From this information, the target population of rice breeders was prepared. The list was subsequently refined several times to ensure all emails were currently active. For some nominations, a second alternative email address was also provided and used to invite participants for the survey. Overall, emails were sent to over 480 rice breeder contacts. The survey was designed to take approximately 15–20 min to complete. Participants were given the option to complete the survey anonymously (the majority of breeders chose to leave their name). However, this does not rule out any potential bias introduced by this non-anonymity. Randomly awarded prizes consisting of IRRI books and souvenirs and a report summarising results were offered as an incentive to complete the survey (for non-anonymous breeders only).

Potential participants—adding up to 315 breeders—were contacted in several rounds through email on behalf of Dr. Bertrand Collard, Senior Scientist and irrigated rice breeder at IRRI at the time of the survey. After the initial email, two independent reminders were sent and an additional 36 people were contacted for the first time whose email address we received during the first survey round. A separate reminder for the breeders in Africa was sent by the Deputy Director General and Director of Research for Development at the Africa Rice Center. A transcript of the actual survey has been included in Additional file 1.

## Results and discussion

The global survey generated a total of 189 responses, leading to a satisfactory response rate of approximately 50%. In this section, we give a general overview of breeder characteristics as displayed by our sample. For ease of reading, all variables are reported in summary tables while graphs are used to highlight some important results. All categorical variables are summarised as counts (*n*). For the categorical variables whose categories sum up to 1, relative frequencies are also given (%). For non-mutually exclusive categorical variables, relative frequencies are not meaningful due to overlapping categories and therefore not given. Means and standard deviations were used to summarise symmetric continuous variables, and medians and interquartile ranges for asymmetric continuous and ordinal variables, where the latter are derived from a Likert scale. For categorical and ordinal variables, levels were indicated in the second column. The number of non-missing observations is given between brackets. To assess the geographic representativeness of the sample, weighting adjustment was applied using the area of paddy rice harvested in 2015. All non-geographic variables, with the exception of the asymmetric continuous variables, were very robust to weighing and did not change significantly. Therefore, the non-weighted variables are presented here. Although this does not rule out bias between more and less progressive breeders within each country, it does indicate that the more progressive countries are not overrepresented. Additional graphs can be found in Additional file 2.

This section includes an overview of some individual breeder characteristics and a discussion of both the internal and external characteristics of breeding organisations. We end with an outline of the surveyed attitudes towards and adoption of rapid generation advance.

### Individual breeder characteristics

Table 1 reports a list of individual breeder characteristics, which can be grouped into personal characteristics, experience, perception towards RGA, operating style and breeding mentality. See Additional file 2: Table 1 for more information about the country of origin distribution for breeders and their highest degree obtained.

**Table 1:** Overview of individual rice breeder characteristics

	Level	All	
*Personal characteristics*			
Age (years) (189 obs.), mean (sd)		45.16	(9.52)
Gender (189 obs.), *n* (%)	Male	148	(78%)
	Female	41	(22%)
Degree (187 obs.), *n* (%)	Bachelor’s degree	8	(4%)
	Master’s degree	67	(36%)
*Experience*	Ph.D.	112	(60%)
Awareness RGA (189 obs.), *n* (%)		163	(86%)
Awareness of benefits RGA (189 obs.), *n* (%)		158	(84%)
Experience breeding (years) (189 obs.), mean (sd)		16.86	(10.02)
Observation RGA (189 obs.), *n* (%)		112	(59%)
Location of observation of RGA (91 obs.), *n*	Observed RGA at IARC	64	
	Observed RGA at NARES	36	
*Perception towards RGA*	Observed RGA at private institute	4	
Percentage reduction in labour estimated from RGA (%) (167 obs.), mean (sd)		43.16	(18.58)
Percentage reduction in land estimated from RGA (%) (171 obs.), mean (sd)		55.62	(21.09)
Reduction time estimated from RGA (years) (114 obs.), mean (sd)		2.39	(1.13)
Certainty of benefits RGA (185 obs.), median [IQR]	1—not certain, 7—very certain	5.00	[4.00, 6.00]
Credibility benefits RGA (185 obs.), median [IQR]	1—not credible, 7—very credible	6.00	[5.00, 7.00]
Feasibility RGA method (184 obs.), median [IQR]	1—not feasible, 7—very feasible	5.00	[5.00, 7.00]
*Operating style*			
Cost minimiser (189 obs.), *n* (%)		134	(71%)
Labour minimiser (189 obs.), *n* (%)		144	(76%)
Actively looking for improvements (189 obs.), *n* (%)		188	(99%)
Risk attitude (189 obs.), median [IQR]	1—avoid risk, 7—like taking risks	5.00	[4.00, 6.00]
Time preference (breeding cycle as obstacle) (189 obs.), median [IQR]	1—not an obstacle, 7—severe obstacle	5.00	[3.00, 6.00]
*Breeding mentality*			
Time needed for fixed lines (crossing) (number of generations) (182 obs.), mean (sd)		7.07	(1.79)
Time needed for fixed lines (trials) (number of generations) (183 obs.), mean (sd)		6.34	(1.07)
Time needed before AYT testing (years) (183 obs.), mean (sd)		2.49	(1.07)

Sample size is 189 observations

*IQR* interquartile range, *AYT* advanced-stage yield trial

First of all, 78% of rice breeders in the survey were male. Looking at the gender gap in science across the developing world, this seems representative. The UNESCO Institute for Statistics (2015) reports the following female employment rates in R&D: 22.6% for East Asia and the Pacific, 18.9% for South and West Asia, and 30.0% for Sub-Saharan Africa. Given these regions represent approximately three quarters of surveyed breeders (see further), 22% of females participating in our survey seem representative. The ages of the rice breeders ranged from 28 to 73 with a mean of 45 years. This is slightly older than the average of 42 years reported by Hargrove [17]. The average age is quite different from the number of years in total breeding experience (mean of 17, range 1–50). In comparison, these numbers do not seem to have changed much since 1978 (mean of 14 years).

**Fig. 2:**
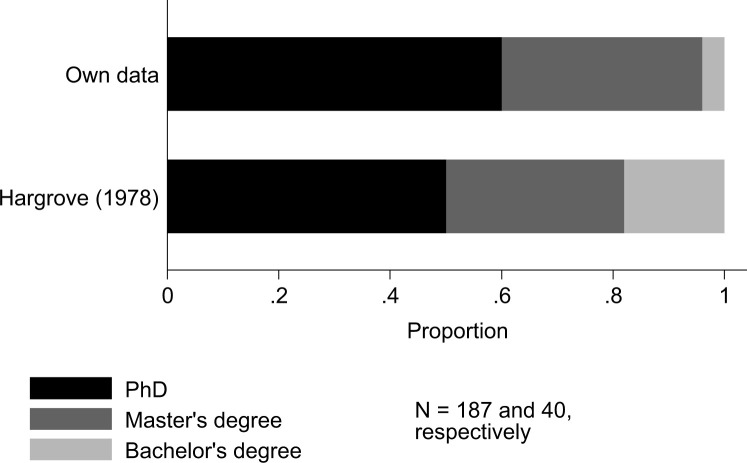
Higher education degrees among rice breeders

**Fig. 3:**
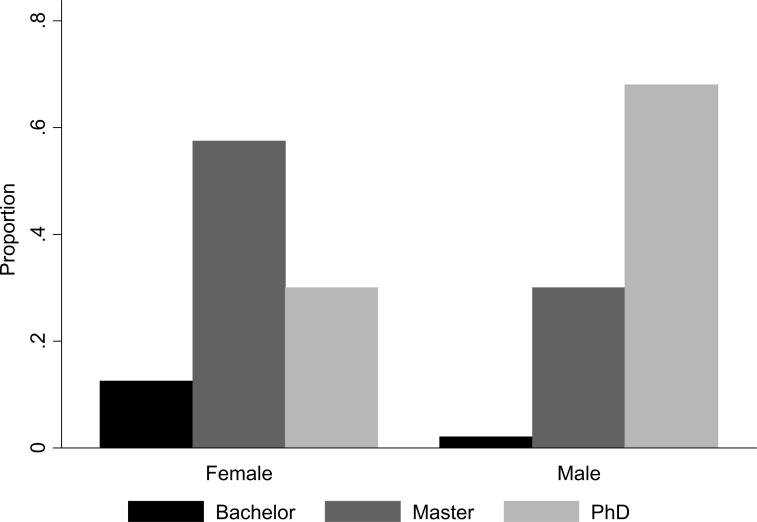
Higher education of rice breeders by gender

In our recent survey more than half of the breeders held a Ph.D. degree, while approximately one-third held a Master’s degree. These numbers are not very different from Hargrove’s [17] survey (Fig. 2). This might indicate the educational landscape might not have changed much over time. However, the location where those PhDs were obtained has changed. Almost 40 years ago, 65% of the PhDs among rice breeders were obtained in highly developed countries (i.e. Canada, the USA, Europe, Japan or Australia) while that number is now only 18%. This seems consistent with the finding that the number of adults with a degree in higher education has been increasing in developing countries with a factor of approximately 2.5 between 1975 and 1990 [33]. In brief, although the distribution of degrees in higher education among rice breeders has not changed much, there does seem to be an increased capacity for tertiary education in rice-growing countries. Lastly, there seems to be a clear gender gap in higher education (Fig. 3). While a majority of males has a Ph.D. and a third only a master’s degree, this balance is reversed for females. This is in line with the World Bank’s observation that the highest gender gaps exist in South Asia, the Middle East and Sub-Saharan Africa—which account for 80% of surveyed breeders [33]. Despite the lack of historical comparison in the rice breeding sector, it is safe to say that improved gender equality in higher education is still a priority in many rice-growing countries.

Since breeding is a relatively slow process, timing and breeders’ perceptions regarding this point are crucially important. To the best of our knowledge, our study is the first one that provides insights into the attitudes of rice breeders regarding breeding cycles (i.e. time from “cross to cross”). Additional file 2: Fig. 2 compares perceptions of time regarding breeding in more detail. We observe that most breeders regard five generations as the absolute minimum time period required to fix lines, both for trials and crossing. This is consistent with theory but surprising because our observations often noted rice breeders who test lines at advanced generations (F10 or beyond) which we believe is unnecessary. Using RGA, breeding lines can easily be fixed in 1–1.5 years, assuming that at least three generations can be completed within 12 months through the use of a greenhouse. The perceived time needed to adequately test elite breeding lines in advanced-stage yield trials before crossing can take place, is considered to be about 2–3 years for the majority of breeders. However, some authors advocate the need for shorter breeding cycles and so the time required for testing in advanced-stage trials could be reduced [1]. In any case, reducing the length of the breeding cycle is considered to be the simplest way to increase the rate of genetic gain.

Regarding operating style, the majority of rice breeders identify themselves as labour and cost minimisers. In fact, hardly any breeder indicated he or she wanted to actively keep the status quo with regard to their breeding method. We also observed that breeders have a relatively high degree of risk taking and see the length of breeding cycle as an obstacle for improving farmers’ livelihoods (time preference; median of 5 on 7-point scale). The latter two factors can be important in determining breeder’s willingness to adopt specific new breeding methods, like RGA.

We also surveyed breeders’ perception towards and experience with the RGA method. We discovered that awareness about RGA is high, both for the method in general as for the benefits it can generate (> 80%). Observation of the RGA method conducted in the field was lower, but still more than half of all breeders had seen the method used before. The majority of breeders observed RGA at an IARC. Most breeders seem confident in the RGA breeding technique; the feasibility of the method and the certainty of the benefits get a median of 5 on a 7-point scale, and the credibility of the projected benefits for the IRRI case even gets a 6 (also, out of 7 points). Breeders also expect to save a significant amount of labour (43%), land (56%) and time (2.4 years). This shows that breeders in general have a very favourable view towards RGA and are well aware of its potential.

### Internal characteristics of organisation

Table 2 reports a list of internal characteristics of the organisation, which can be grouped into type of organisation, breeding operations, job function, freedom and resources.

**Table 2:** Overview of internal characteristics of rice breeding organisations

	Level	All	
*Type of organisation*			
Private institute (189 obs.), *n* (%)		11	(6%)
*Breeding operations*			
Main method (188 obs.), *n* (%)	Bulk	10	(5%)
	Combination	7	(4%)
	Pedigree	147	(78%)
	Other	24	(13%)
Varieties (188 obs.), *n* (%)	Hybrids only	13	(7%)
	Inbreds only	130	(69%)
	Inbreds and Hybrids	45	(24%)
Experience main method (years) (189 obs.), mean (sd)		13.93	(9.43)
*Job function*			
Permanent employment contract (189 obs.), *n* (%)		168	(89%)
Hierarchy (177 obs.), *n* (%)	Head of department	65	(37%)
	Report directly to head of department	100	(56%)
	Report indirectly to head of department	12	(7%)
*Freedom*			
Opportunities for new techniques (189 obs.), *n* (%)		175	(93%)
Permission to adopt RGA (153 obs.), *n* (%)		149	(97%)
*Resources*			
Timing labour constraint (185 obs.), *n*	Flowering	20	
	Harvesting	135	
	Land preparation	52	
	Post-harvest	67	
	Seedling nursery	46	
	Transplanting	128	
	Vegetative stage	21	
	Never	13	
Severity labour constraint (189 obs.), median [IQR]	1—not severe, 7—prohibiting operations	4.00	[3.00, 6.00]
Staff institute (168 obs.), median [IQR]		100.00	[27.50, 355.00]
Staff department (173 obs.), median [IQR]		17.00	[10.00, 40.00]
Staff team (174 obs.), median [IQR]		6.00	[5.00, 10.00]
Labour workers harvesting (174 obs.), median [IQR]		10.00	[5.00, 20.00]
Labour workers seedling/transplanting (170 obs.), median [IQR]		10.00	[5.00, 20.00]
Problems in cash flow (176 obs.), *n* (%)		115	(0.65)
Budget size (10,000 US$) (141 obs.), median [IQR]		2.56	[1.00, 12.72]
Likelihood budget cut (152 obs.), *n* (%)	Likely	53	(35%)
	Possibly	71	(47%)
	Not likely	28	(18%)
Land available (ha) (171 obs.), median [IQR]		3.00	[1.50, 6.00]
Greenhouse present (189 obs.), *n* (%)		130	(69%)

Sample size is 189 observations

*IQR* interquartile range

Public breeding institutes dominate as only 6% of surveyed breeders are active in a private breeding station. This reflects that the majority of rice breeding currently occurs in the public sector which is consistent with the prevalence of inbred varieties, although hybrid rice is common in some countries like China and gaining popularity in other countries. Hybrid varieties are usually produced by private companies, due to the requirement to purchase seeds for every season. Looking at the type of varieties bred, almost 70% of breeders use inbreds exclusively, and less than 10% work on hybrid rice. However, the number of breeders working on hybrid rice would have been higher if a larger sample from China was obtained.

**Fig. 4:**
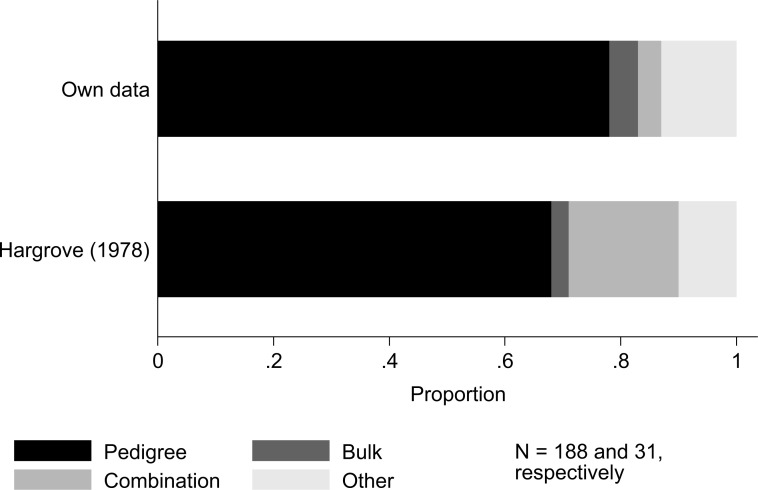
Overview of rice breeding methods used

Regarding breeding methods, the classic pedigree method is still by far the most common breeding method; almost 80% of breeders still use it as their main breeding method based on our results (Fig. 4). This method was widely popularised as the most effective rice breeding method at IRRI during the 1970s and 1980s [19, 21]. Comparing with Hargrove [17], we see this number has not changed much over time. This is an interesting result as the classical pedigree method has been used for many decades. Adding to this, breeders’ experience with their main breeding technique (mean of 14, range of 1–50) is very close to breeders’ total experience (mean of 17, range of 1–50; see also Additional file 2: Fig. 1). This might indicate that breeders in general do not shift to another breeding method easily.

In the agricultural science literature, much attention goes to farmers and how their characteristics influence adoption of technologies. However, farm structures are not comparable to the large organisational structures displayed by breeding institutes. These organisational differences are most likely to influence the adoption process. In our survey, we see that breeders still enjoy a relatively high degree of freedom as the large majority have a permanent employment contract (89%), expect to get permission to adopt RGA (97%) and have opportunities to experiment with and implement new breeding techniques (93%). About one-third of respondents are heads of departments and slightly more than half report directly to the head of department. These high percentages might point to the fact that breeders themselves enjoy a relatively high degree of decision-making power.

As mentioned above, NARES are increasing their capacity in performing their own breeding operations. Nevertheless, resource constraints remain a key problem for many breeders including those at IARC research centres and even private breeding companies. A greenhouse or screenhouse facility (which is generally recommended for the RGA procedure) is relatively common (69%) but so are problems in cash flows: 65% of breeders indicated there are times of the year where their budget is insufficient. Thus, breeding methods requiring a greenhouse might not be a problem as long as they do not introduce (unforeseen) additional cash expenses during the year. Other important resources are labour, budget and area of land available. Starting with the first, the median number of staff depends on the level of aggregation: institutes employ around 100 staff members, departments about 17 and breeding teams around 6. It is important to note though that the variation in staff members is much larger for institutes than for the lower levels (departments and teams). While a large number of institutes employ relatively low levels of staff, there is a non-trivial number that employs 1000 or more staff members (see Additional file 2: Fig. 3). This large variability is likely to inflate the mean somewhat, and therefore the median might be a better indicator of the overall employment levels of staff across breeding institutes. Additionally, although clearly specified, the inclusion of seasonal labourers might be an explanation for these rather high levels of staff employment for certain institutes. Next, during labour peaks (i.e. harvesting and seeding/transplanting), a median number of 10 labour workers are employed per breeding team.

**Fig. 5:**
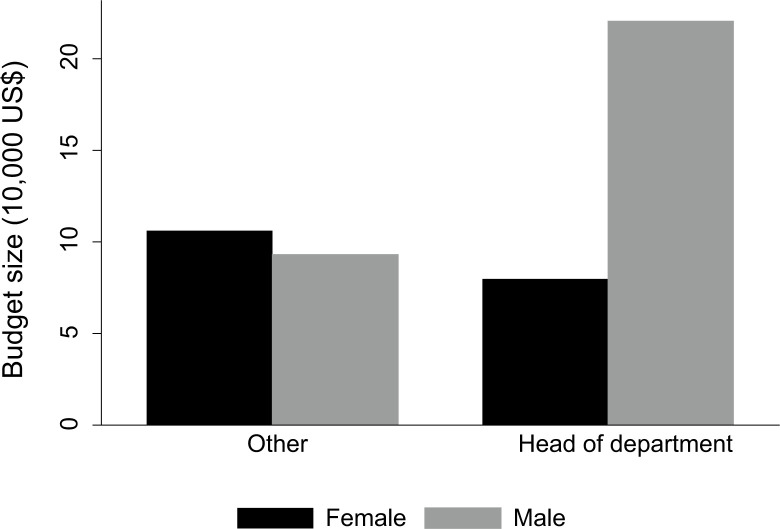
Comparison of rice breeders’ budgets among heads of department by gender

Other important resources in the breeding process are budget and land available. Looking at the median, each breeding institute uses around 3.00 ha of land and US$ 25,609. Again, these variables portray a very high variability: the area of land ranges between 50 m^2^ and 267 ha (13 breeders dispose of an area over 20 ha)^1^ and the available budget ranges between US$ 47 and US$ 3,350,000 (14 breeders dispose of budgets over US$ 400,000)^2^ (see Additional file 2: Fig. 4). Despite the presence of these remarkably high values, there is a clear concentration of institutes at relatively low values (indicated by the median being closer to the 25th than the 75th percentile). Thus, although the total number of resources seems quite abundant, this impression is biased due to the presence of some relatively resource-rich institutes in the survey. Many smaller institutes may actually lack the necessary resources to innovate their breeding operations and perform their current breeding goals. Approximately half of the breeders even think a budget cut within the next 5 years is possible while 35% think it is likely. Lastly, gender also plays a role in determining budgets. Females who are head of their department dispose of only one-third of the budget their male counterparts dispose of (Fig. 5). It is interesting to note that this gender gap does not seem to hold at a lower hierarchical level.

### External characteristics of organisation

**Fig. 6:**
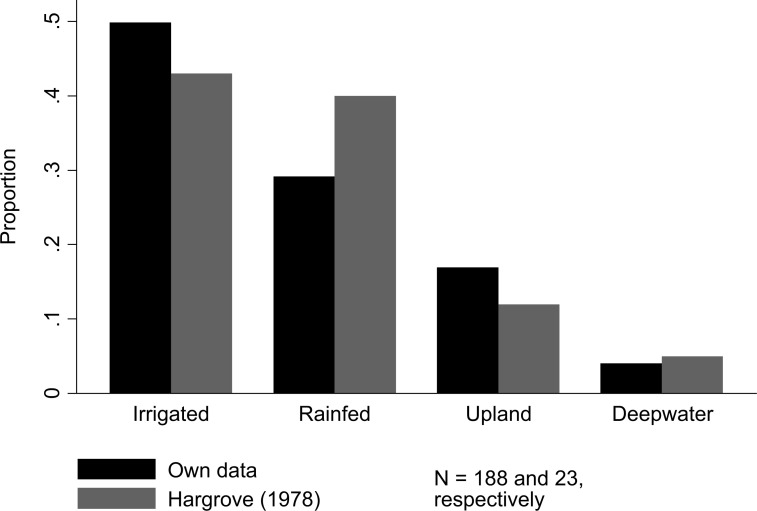
Proportion of ecosystems in which rice breeders are operating

Table 3 reports a list of external characteristics of the organisation, which can be grouped into environment and geography. We observed that almost 80% of breeders work under irrigated conditions, which is half of all ecosystems reported by Hargrove [17]. Approximately 30% of ecosystems reported by Hargrove [17] are rainfed and 17% upland. These numbers are consistent with Hargrove’s [17] survey (Fig. 6). Additionally, approximately 12% of breeders focus on temperate environments, and 29% focus on coastal, flood-prone or both regions. The average number of seasons per year was 1.6.

**Table 3:** Overview of external characteristics of rice breeding organisations

	Level	All	
*Environment*			
Ecosystem (188 obs.), *n*	Coastal	25	
	Deepwater	12	
	Flood prone	30	
	Irrigated	147	
	Rainfed	86	
	Temperate	23	
	Upland	50	
Seasons per year (189 obs.), mean (sd)		1.62	(0.56)
*Geography*			
Origin (location) institute (189 obs.), *n* (%)	Asia	123	(65%)
	Africa	28	(15%)
	South America	23	(12%)
	North America	8	(4%)
	Europe	2	(1%)
	Middle East	5	(3%)

Sample size is 189 observations

Our focus was on breeders worldwide although, as can be expected, most rice breeders are active in breeding stations in Asia (65%) with 15% of breeders active in Africa and 12% in South America. Rice production in Europe is extremely limited; only Russian breeders responded to our survey (other countries where rice is grown include Italy, Spain, Greece, Portugal, France, Romania, Bulgaria and Hungary). Unfortunately, the IRRI network in Europe was limited because of IRRI’s few collaborations in rice breeding with European countries.

### Adoption of and willingness to adopt (WTA) rapid generation advance

Table 4 summarises the current adoption status of RGA among breeders and their stated willingness to adopt RGA, as well as underlying reasons for those decisions.

**Table 4:** Rice breeders’ adoption status/intentions and underlying reasons regarding RGA

	Level	All	
Adoption and Willingness to Adopt (WTA) (184 obs.), *n* (%)	No adoption, but WTA for testing	28	(15%)
	No adoption, but WTA to secondary method	51	(28%)
	No adoption, but WTA to main method	23	(13%)
	No adoption nor WTA	5	(3%)
	Adoption for testing, but WTA to secondary method	18	(10%)
	Adoption for testing, but WTA to main method	6	(3%)
	Adoption for testing, but no further WTA	2	(1%)
	Adoption as secondary method, but WTA to main method	31	(17%)
	Adoption as secondary method, but no further WTA	12	(7%)
	Adoption as main method	8	(4%)
Reasons for willingness to adopt RGA (157 obs.), *n*	Time saving	140	
	Cost reduction	120	
	Labour reduction	120	
	Land reduction	118	
	Genetic gain	57	
	Public benefits	27	
	Subsidies	3	
Destination of resource savings when willing to adopt RGA (157 obs.), *n*	Extra screening	89	
	Extra crosses	75	
	Multilocation yield trails (MLYT)	72	
	Larger yield trials	51	
	Nothing	4	
Reasons for non-willingness to adopt RGA (19 obs.), *n*	No greenhouse/screenhouse	6	
	Lack of money	2	
	Need approval from superiors	2	
	Not enough labour available	3	
	Not certain about benefits	6	
Obstacles when willing to adopt RGA (157 obs.), *n*	No greenhouse/screenhouse	109	
	Lack of money	67	
	Not enough labour available	24	
	Need approval from superiors	32	
	Not certain about benefits	19	

Sample size is 189 observations in total, 157 for those willing to adopt and 19 for those not willing

**Fig. 7:**
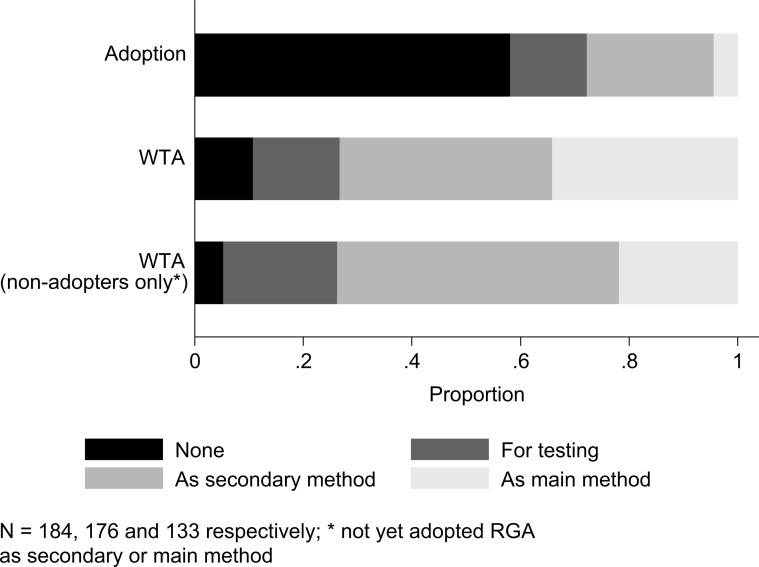
Rice breeders’ adoption status/willingness to adopt RGA

To survey willingness to adopt (WTA), we presented breeders with a brief scenario of RGA operations at the International Rice Research Institute (IRRI). Afterwards we asked breeders whether they were willing to adopt RGA, if they had not done so already. We already noted that breeders showed a very favourable view towards RGA. Looking at Table 4, we see this was reflected in a high level of WTA. Of all breeders who had not yet adopted RGA as their main method, approximately 75% were willing to adopt RGA as their secondary or main method; only 10% did not want to adopt RGA in any way, not even for testing (Fig. 7). This figure remains the same if we look only at those who had not yet adopted RGA as secondary or main method. Both the positive view and high WTA are in sharp contrast with the low degree of adoption. Only a quarter used RGA as a secondary method and approximately 4% as their main breeding method. The use of a secondary method was interesting (i.e. that breeders use multiple methods) and could have been due to the requirement to develop some populations for research purposes (e.g. genetic analysis) rather than actual breeding. It should also be noted that in practice, it is complicated to introduce large-scale changes to breeding operations.

When asked why people were not willing to adopt, the lack of a greenhouse and no certainty about the benefits were given most often in reply. Note that the low number of responses was due to the fact that only 19 breeders were not willing to adopt RGA in any way (excluding main adopters). For those willing to adopt, the lack of a greenhouse and lack of money were seen as the biggest obstacles. For these people, time savings and reductions in cost, labour and land were all given as important reasons for adopting RGA. Among breeders reporting only one obstacle to adopting RGA, a lack of greenhouse infrastructure was given in more than half of the cases (see Additional file 1). When asked for the use of saved resources, extra screening was mentioned as the most common answer in our survey, but also extra crosses, yield trials at multiple locations and larger yield trials were reported. Regarding crosses, it is interesting to note that some previous research suggests that making extra crosses does not improve breeding efficiency [34]. This could point to Cleveland’s [9] observation that plant breeding science is both a biophysical and social construction of reality. Only a small minority would not use the saved resources at all. These observations led us to hypothesise that these reasons were based on subjective views rather than evidence and that research involving the optimisation of rice breeding programmes is a research gap that warrants further investigation.

Interestingly, no breeder commented on the possible use of DNA markers; however this was not listed as an option (i.e. but it could have been listed as ‘other’). We deliberately did not include survey questions about the use of DNA marker technology because in our experience, the current use of molecular breeding in routine rice breeding is still very low in most countries. Furthermore, including additional questions to survey this topic would have greatly increased the length of the survey and would have greatly widened the scope of our study, and may have diminished our main objectives. It is worth noting that molecular breeding schemes such as marker-assisted selection or genomic selection can be implemented in any breeding scheme, but may specifically provide further improvements in efficiency when incorporated in a RGA-based breeding scheme [10].

## Conclusion

In this article, we have displayed a range of characteristics describing breeders’ characteristics, attitudes and adoption behaviour towards new breeding technologies. To cover the entire spectrum of characteristics, we looked at individual breeder characteristics, as well as the internal and external characteristics of the organisation. This way we have presented a relatively unique insight into the population of rice breeders worldwide. Overall, rice breeders are highly educated and have a long experience with their main method. A clear gender gap regarding education seems present. The variability in resources (staff, land and budget) is particularly large and makes it hard to assess to overall operational capacity of rice breeders worldwide. While some institutes clearly have access to large resources, these need to be considered exceptions given the large number of resource-poor institutes. Most breeders are active in irrigated conditions. Contrary to expectations, breeders expressed a relatively high degree of operational freedom and relying on their stated operating styles, rice breeders seem relative open to innovation. Our results permitted us to characterise global rice breeders, especially in Asia, Africa and South America. To the best of our knowledge, this was not done before and allows us to lay down a reference about the characteristics and adoption behaviour of rice breeders. As a baseline, it will be crucial for comparisons in the future. By identifying the global rice breeder population, we have made it visible for the first time. Where possible, we compared our results to previous ones and overall our findings seem consistent and do not differ much for the variables reported.

Secondly, we looked at several characteristics related to rapid generation advance. Overall, our findings indicated a positive perception towards rapid generation advance (RGA). Most breeders have an operating style with a relatively high degree of risk taking and time preference, greenhouses were not uncommon (69%) and almost all breeders expected to get permission to adopt RGA (97%). Approximately 85% of breeders are aware of RGA and its benefits. More than half of breeders have observed the actual use of RGA. Furthermore, breeders are confident in the RGA technique (high feasibility, certainty and credibility of benefits) and estimate its resource savings to be substantial. As a consequence, breeders’ willingness to adopt RGA was remarkably high: only 10% did not want to adopt RGA in any way, not even for testing. Surprisingly, adoption of RGA remains low (4% as main method) while the classical well-known pedigree breeding method is still the most common breeding method. Possible reasons are lack of greenhouse facilities, uncertainty about the benefits among breeders and insufficient operational budgets. Our results could be useful to develop targeted extension material or interventions for implementing new technologies, which could be useful to high-level agricultural managers, international research centres and aid agencies. These technologies could have tremendous impacts in terms of accelerating the delivery of rice varieties that are targeted to the needs of rice farming communities and consumers around the world.

## Data Availability

The dataset generated and analysed during the current study is not publicly available due to pending research articles relying on the same dataset but is available from the corresponding author upon request.

## Footnotes

1 Under normal circumstances, 50 m² is inadequate as a breeding area. In this case, the breeder in question uses his/her breeding area solely for experimenting with rice under conditions that are generally unfavourable to rice.

2 Under normal circumstances, US$ 47 is inadequate as a breeding budget. In this case, the small dollar value might be due to a serious devaluation of the Venezuelan bolivar.

## Additional files

**Additional file 1**. Transcript of the actual survey.

**Additional file 2**. Additional tables and figures from the same dataset discussed in this article.
