# Supplementary material for: Global survey of rice breeders to investigate characteristics and willingness to adopt alternative breeding methods
Source: Agric Food Secur. 2018 Jun 25;7:40. doi: 10.1186/s40066-018-0191-3 (PMC7507798; doi:10.1186/s40066-018-0191-3)
Supplement: Supplementary file 1 [file AFS-2018-s40066-018-0191-3-S1.pdf]

IRRI Breeders' Survey on Rapid Generation Advance (RGA)

*Required

Welcome to IRRI Breeders' Survey on Rapid Generation Advance (RGA)

Thank you for agreeing to participate in this survey on currently used rice breeding methods. We are also trying to evaluate the level of interest of adoption of rapid generation advance (RGA) or single seed descent in the future. All answers you provide will be kept confidential. This survey should take only 10-15 minutes to complete. Please click ‘Continue’ to begin the survey.

General characteristics

1. **What is your name?**
   You can leave this open if you want to answer the questionnaire anonymously. Be aware that leaving this field open, disables you from receiving any prizes.

   ....

2. **What is your year of birth?** *

   ....

3. **What is your gender?** *
   Mark only one oval.

   ◯ Male
   ◯ Female

4. **What is your country of origin?** *

   ....

5. **What is the highest degree you obtained?** *
   We refer to studies related to breeding.
   Mark only one oval.

   ◯ Bachelor's degree
   ◯ Master's degree
   ◯ PhD
   ◯ Other: ....

6. **In what country did you obtain this highest degree?** *

7. **How many years of experience do you have in breeding?** *
   Please use numbers, not letters

8. **What is the name of your institute?** *

9. **In which country is your institute located?** *

10. **What is your job function exactly at your institute?** *
    For example: senior breeder

11. **What is the length of your employment contract** *
    Mark only one oval.
    - Permanent
    - 3 years
    - 4 years
    - 5 years
    - Other:

12. **What is your main breeding method?** *
    Mark only one oval.
    - Pedigree
    - Bulk
    - Modified pedigree
    - Modified bulk
    - Rapid generation advance
    - Other:

13. **If you have comments about the previous question, please write them below.**

....

....

....

....

....

14. **How many years have you been using this breeding method?** *

Please use a number, not letters.

....

15. **After which generation do you consider your breeding lines to be fixed (enough) so that field trials can take place?** *

Mark only one oval.

- F5
- F6
- F7
- F8
- F9
- F10
- Other: ....

16. **At which generation do you consider fixed lines to be suitable for using in crossing?** *

Mark only one oval.

- F5
- F6
- F7
- F8
- F9
- F10
- F11
- F12
- Other: ....

17. **How many years of testing in advanced stage yield trials (e.g. replicated yield trials) do you consider to be necessary before using in crossing?** *
Mark only one oval.

- ( ) 1
- ( ) 2
- ( ) 3
- ( ) 4
- ( ) 5
- ( ) Other: ....

18. **What types of varieties do you breed?** *
Multiple answers possible.
Tick all that apply.

- [ ] Inbreds
- [ ] Hybrids

19. **In what type of ecosystem do you work?** *
Multiple answers possible.
Tick all that apply.

- [ ] Irrigated
- [ ] Rainfed
- [ ] Temperate
- [ ] Flood-prone
- [ ] Coastal
- [ ] Upland
- [ ] Deepwater
- [ ] Other: ....

Rapid Generation Advance

We are going to describe details about the Rapid Generation Advance method. Please read carefully before going to the questions.

Rapid Generation Advance

Rapid Generation Advance (RGA) or synonymously Single Seed Descent (SSD) is an alternative breeding method to the widely used pedigree method used in self-pollinated crops. Two main objectives in breeding self-pollinated crops are: (1) the generation of fixed lines after hybridisation and (2) the selection of agronomically superior lines from segregating populations. In the pedigree method, these two functions are performed simultaneously. The RGA method separates line fixation and selection steps.

This method is usually applied to F2 breeding populations and continued until the F5 or F6 generation, once lines are homozygous or fixed. Instead of growing plants in the field, plants are grown in a screen- or greenhouse to control conditions. Due to a higher seeding density, restricted soil volume and/or reduced fertiliser application, plants mature earlier but produce only a few seeds per plant. No selection takes place until after the F5 generation to speed up the process of developing fixed lines (i.e. inbreeding for homozygosity). After the screenhouse, a seed increase step in the field is needed to produce enough seeds prior to testing in observational yield trials. During this step, lines are selected based on agronomic traits for the first time. In summary, the RGA method consists of growing at least 4

generations in a screenhouse plus one generation for seed increase in the field.

There are four important benefits of using RGA. First RGA saves substantial land area required for growing breeding populations compared to other breeding methods. Secondly labour can be significantly reduced. Thirdly, materials and tools used for data collection are significantly lower. Finally, during RGA, plants can be manipulated so that they mature earlier usually permitting extra generations to be grown each year compared to field-grown plants. This saves at least one to two years over the entire breeding process, potentially leading to quicker variety release. An essential requirement for the RGA method is a screenhouse or greenhouse, together with some smaller inputs like small pots or seedling trays.

The disadvantages of using RGA are: (1) that the identity of individual F2 plants is lost, (2) desirable plants may be lost due to genetic drift or random factors, and that (3) unselected lines are retained until the first field testing step.

By the end of both pedigree and RGA methods, at least 1000 breeding lines are produced for inclusion in the observational yield trial.

At IRRI in 2012, a RGA-based breeding method was implemented. This breeding scheme uses a greenhouse of approximately 700 m² to generate at least 1000 new breeding lines for inclusion in a observational yield trial. This scheme showed a reduction in land of approximately 70% and a reduction in seasonal labour of approximately 55% compared to a typical pedigree breeding program.

Steps of the RGA process

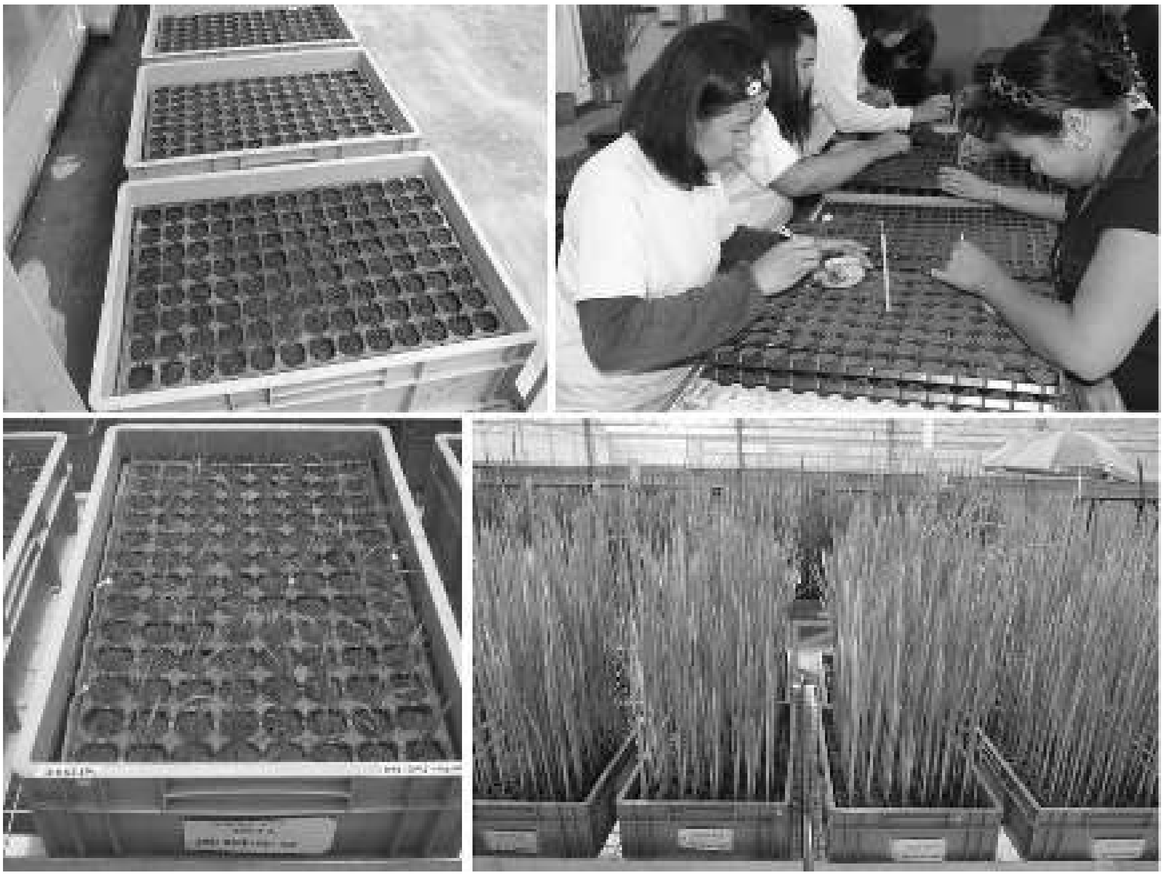

Current RGA facility at IRRI

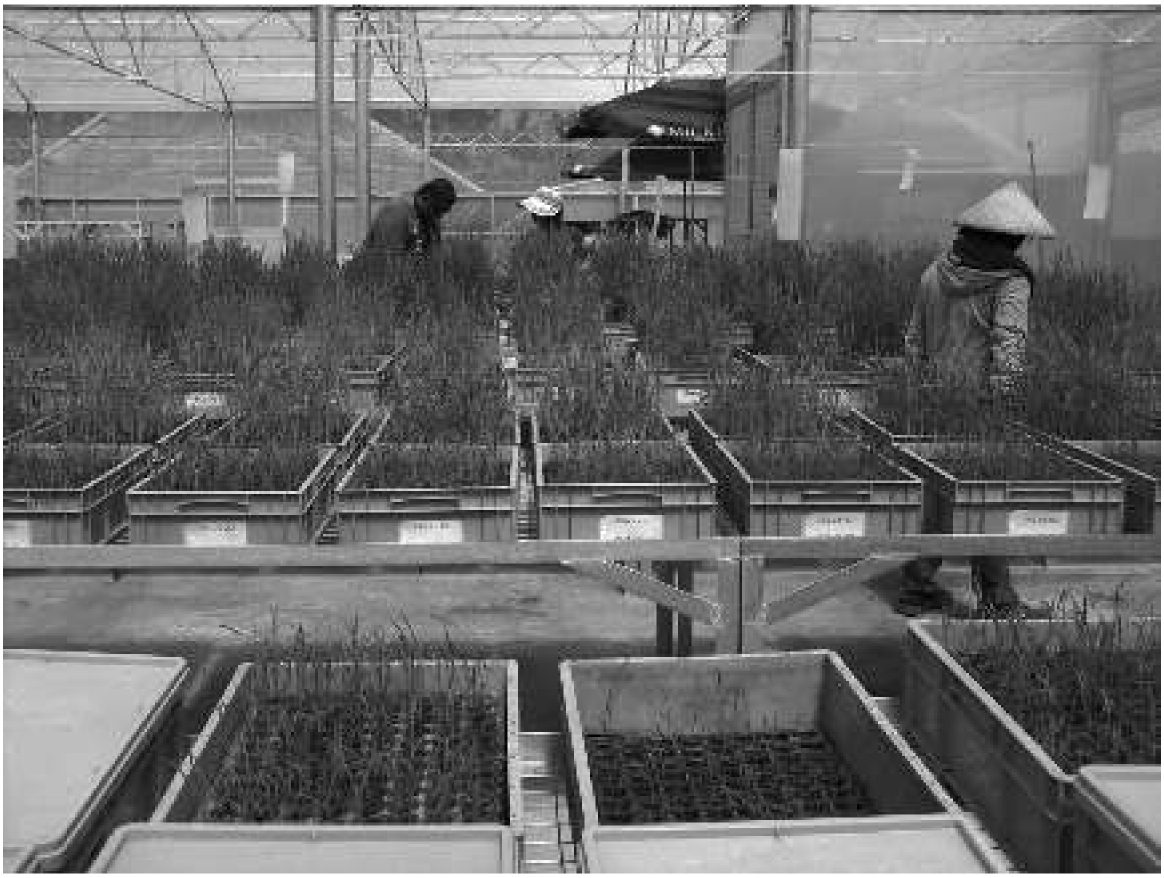

Comparison of time required for pedigree and RGA breeding methods

Year	Season	Pedigree		RGA	
		Slow	Fast	Slow	Fast
1	Dry	Hybridization	Hybridization	Hybridization	Hybridization
	Wet	F1	F1	F1	F1
2	Dry	F2	F2	F2-F4	F2-F5
	Wet	F3	F3		
3	Dry	F4	F4	F5	F6*
	Wet	F5	F5	F6*	
4	Dry	F6	F6		
	Wet	F7	F7*		
5	Dry	F8*			
	Wet				

*: Seed multiplication step

Rapid Generation Advance

20. **Had you ever heard about RGA before?** *

That is: before reading our introction.

Mark only one oval.

- Yes
- No *Skip to question 24.*

Rapid Generation Advance

21. **Had you ever heard about the benefits of using RGA before?** *

That is: before reading our introduction.

Mark only one oval.

- Yes
- No

22. **Have you observed RGA in a screenhouse/greenhouse or in the field before?** *

Mark only one oval.

- Yes *Skip to question 23.*
- No *Skip to question 24.*

Rapid Generation Advance

23. **Where have you observed RGA in practice before?** *

Tick all that apply.

- International Rice Research Institute (IRRI)
- National agricultural research station
- International agricultural research station
- University agricultural research station
- Government agricultural research station
- Other: ....

Rapid Generation Advance

24. **How feasible do you think the RGA method is for rice breeding?** *

Mark only one oval.

	1	2	3	4	5	6	7	
Not feasible								Very feasible

25. **If you have comments about the previous question, please write them below.**

....
....
....
....
....

26. **How credible do you think the benefits mentioned in the scenario are?** *
Mark only one oval.

	1	2	3	4	5	6	7	
Not credible	◯	◯	◯	◯	◯	◯	◯	Very credible

27. **If you have comments about the previous question, please write them below.**

....
....
....
....
....

28. **What would be the estimated labour reduction you could achieve in your program by adopting RGA?**
You can give an estimation in %.

....

29. **What would be the estimated land reduction you could achieve in your program by adopting RGA?**
You can give an estimation in %.

....

30. **What would be the estimated time savings you could achieve in your program by adopting RGA?**
You can give an estimation in years.

....

31. **How certain do you think the benefits of RGA are?** *
Mark only one oval.

	1	2	3	4	5	6	7	
Very risky/uncertain	◯	◯	◯	◯	◯	◯	◯	Certain

32. **Do you think your superiors would allow you to adopt RGA in the future?** *
Mark only one oval.

- Yes
- No
- Don't know

33. **Have you ever used RGA before or are you currently using RGA?** *
Mark only one oval.

- No *Skip to question 34.*
- Yes, for testing *Skip to question 35.*
- Yes, using as a secondary breeding method *Skip to question 37.*
- Yes, using as main breeding method *Skip to question 39.*

Rapid Generation Advance

34. **Would you be willing to adopt RGA based on the scenario given above?** *
Mark only one oval.

- No *Skip to question 46.*
- Yes, but only for testing *Skip to question 40.*
- Yes, but only as a secondary breeding method *Skip to question 40.*
- Yes, as main breeding method *Skip to question 40.*

Rapid Generation Advance

35. **What were your reasons for testing RGA (before)?**

....

....

....

....

....

36. **Would you be willing to adopt RGA as a breeding method based on the scenario given above?** *
Mark only one oval.

- No *Skip to question 46.*
- Yes, but only as a secondary breeding method *Skip to question 40.*
- Yes, as main breeding method *Skip to question 40.*

Rapid Generation Advance

37. **What were your reasons for adopting RGA as a secondary breeding method before?**

....

....

....

....

....

38. **Would you be willing to adopt RGA as main breeding method based on the scenario given above?** *

Mark only one oval.

- ◯ No *Skip to question 46.*
- ◯ Yes *Skip to question 40.*

Rapid Generation Advance

39. **What were your reasons for adopting RGA as main breeding method before?**

....

....

....

....

....

Skip to question 48.

Rapid Generation Advance

40. **What are the main reasons for this decision?** *

Multiple answers possible.
Tick all that apply.

- ☐ Cost reduction
- ☐ Time saving
- ☐ Labour reduction
- ☐ Land reduction
- ☐ More public benefits
- ☐ Subsidies
- ☐ Increasing rate of genetic gain

41. **If you have comments about the previous question, please write them below.**

....

....

....

....

....

42. **What would you consider doing with the savings in resources?** *

Tick all that apply.

- [ ] Extra crosses
- [ ] Larger yield trials
- [ ] Yield trials at multipe locations
- [ ] Extra screening
- [ ] Nothing
- [ ] Other: ....

43. **If you have comments about the previous question, please write them below.**

....

....

....

....

....

44. **What in your opinion would be the biggest obstacles for adopting this method?** *

Multiple answers possible.

Tick all that apply.

- [ ] Lack of money
- [ ] No greenhouse/screenhouse
- [ ] Not enough labour availabe
- [ ] Need approval from superiors
- [ ] Not certain about benefits
- [ ] Other: ....

45. **If you have comments about the previous question, please write them below.**

....

....

....

....

....

Skip to question 48.

Rapid Generation Advance

46. **What are the main reasons for this decision?** *

Multiple answers possible.
Tick all that apply.

- [ ] Lack of money
- [ ] No greenhouse/screenhouse
- [ ] Not enough labour availabe
- [ ] Need approval from superiors
- [ ] Not certain about benefits
- [ ] Other: ....

47. **If you have comments about the previous question, please write them below.**

....

....

....

....

....

Skip to question 48.

Background

48. **Is there opportunity for you to implement new techniques in your breeding method?** *

Mark only one oval.

- ( ) Yes
- ( ) No

49. **What is your function relative to the head of the breeding department?** *

Mark only one oval.

- ( ) I am head of the breeding department
- ( ) I report to the head of the breeding department
- ( ) I report to someone who reports to the head of the breeding department
- ( ) Don't know
- ( ) Other: ....

50. **Are you actively looking for ways to improve your breeding program?** *

Mark only one oval.

- ( ) Yes
- ( ) No

51. **How do you see yourself as a decision maker: how likely will you take risks when choosing breeding methods?** *
Mark only one oval.

	1	2	3	4	5	6	7	
Avoid taking risks	⬭	⬭	⬭	⬭	⬭	⬭	⬭	Like taking risks

52. **If you have comments about the previous question, please write them below.**

....
....
....
....
....

53. **Does your institute have a greenhouse or screenhouse facility?** *
Mark only one oval.

- ⬭ Yes
- ⬭ No

54. **Approximately how many staff work in the institute?**
We refer to staff as everyone who is not a seasonal worker. Please use a number, not text.

....

55. **Approximately how many staff work in the breeding department?**
We refer to staff as everyone who is not a seasonal worker. If your institute only has a breeding department and no other departments, you can fill in the same answers as for the previous question. Please use a number, not text.

....

56. **Approximately how many staff work in your team?**
We refer to staff as everyone who is not a seasonal worker. Please use a number, not text.

....

57. **Approximately how many people work as contract workers during seeding/transplanting for your team?**
We refer to contract workers as everyone who is a seasonal worker during labour peaks. Please use a number, not text.

....

58. **Approximately how many people work as contract workers during harvesting for your team?**
We refer to contract workers as everyone who is a seasonal worker during labour peaks. Please use a number, not text.

....

59. **What is the severity of the labour constraints you face?** *
This question refers to labour during seasonal peaks.
Mark only one oval.

	1	2	3	4	5	6	7	
No labour constraints	⬭	⬭	⬭	⬭	⬭	⬭	⬭	Severe labour constraints prohibiting operations

60. **When do you face labour constraints?** *
This question refers to labour during seasonal peaks. Multiple answers are possible.
Tick all that apply.

- ☐ Land preparation
- ☐ Seedling nursery
- ☐ Transplanting
- ☐ Vegetative stage
- ☐ Flowering
- ☐ Harvesting
- ☐ Post-harvest
- ☐ Never
- ☐ Other: ....

61. **Do you plan to reduce the labour of your breeding operation?** *
Mark only one oval.

- ⬭ Yes
- ⬭ No

Background

62. **What is the area used by your breeding operation?**
You can answer this question in ha, square meters, acres, square feet or another unit, but please specify the units in the next question.

....

63. **Please specify the units you used in the previous question.**
Mark only one oval.

- ( ) Hectares (ha)
- ( ) Sqare meters (m^2)
- ( ) Acres (ac)
- ( ) Square feet (sq ft)
- ( ) Other: ....

64. **How many seasons a year do you conduct nursery breeding or field trials in a typical year?** *
Mark only one oval.

- ( ) 1
- ( ) 2
- ( ) 3

65. **To which extent do you think the length of your breeding cycle is an obstacle for improving farmers' livelihood?** *
We refer to the time it takes from cross to the submission of a new potential variety to the national testing system.
Mark only one oval.

	1	2	3	4	5	6	7	
No obstacle	( )	( )	( )	( )	( )	( )	( )	Severe obstacle

66. **If you have comments about the previous question, please write them below.**

....

....

....

....

....

67. **Do you face problems in cash flow?** *

Meaning: times of the year without sufficient budget to purchase or pay for necessary bills.

Mark only one oval.

- Yes
- No
- Don't know

68. **What is your expectation regarding budget cuts within the next 5 years?** *

Mark only one oval.

- Not likely
- Possibly
- Likely
- Don't know

69. **What is the size of your annual budget?**

You can give a number in your own currency or a foreign currency (e.g. dollars, euro), but please specify the units in the next question.

....

70. **Please specify the units you used in the previous question.**

....

71. **Do you plan to reduce the costs of your breeding operation?** *

Mark only one oval.

- Yes
- No

72. **If you like to receive a report with the summarised results of this study, you can leave your email address below.**

This information will be kept confidential and will not be given to others.

....

Powered by
Google Forms
