# Supplementary material for: Global survey of rice breeders to investigate characteristics and willingness to adopt alternative breeding methods
Source: Agric Food Secur. 2018 Jun 25;7:40. doi: 10.1186/s40066-018-0191-3 (PMC7507798; doi:10.1186/s40066-018-0191-3)
Supplement: Supplementary file 2 [file AFS-2018-s40066-018-0191-3-S2.pdf]

Global survey of rice breeders to investigate characteristics and willingness to adopt alternative breeding methods

Bert Lenaerts[1,2,3*], Bertrand C.Y. Collard[4] and Matty Demont[2]

[1] Centre for Environmental Sciences, UHasselt, Hasselt, Belgium

[2] Agri-food Policy Platform, International Rice Research Institute (IRRI), Los Baños, Philippines

[3] Department of Earth and Environmental Sciences, KU Leuven, Leuven, Belgium

[4] Plant Breeding Platform, International Rice Research Institute (IRRI), Los Baños, Philippines

[*] Corresponding author: m.demont@irri.org

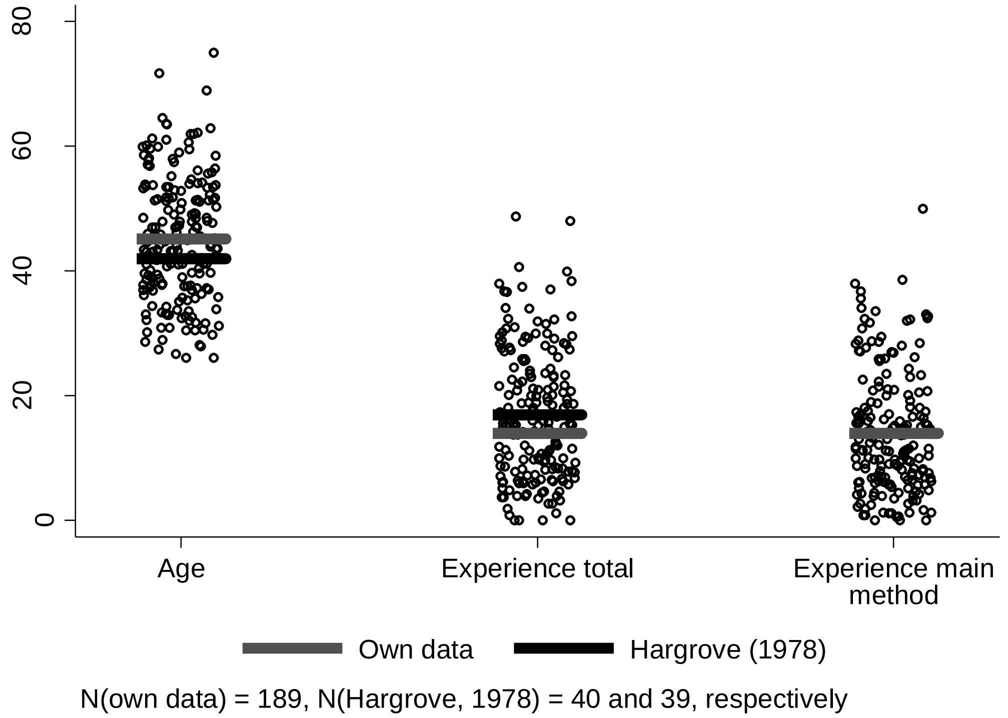

Fig. 1 Dot plot of age, experience as breeder and experience with their main breeding method. The horizontal lines represent the means.

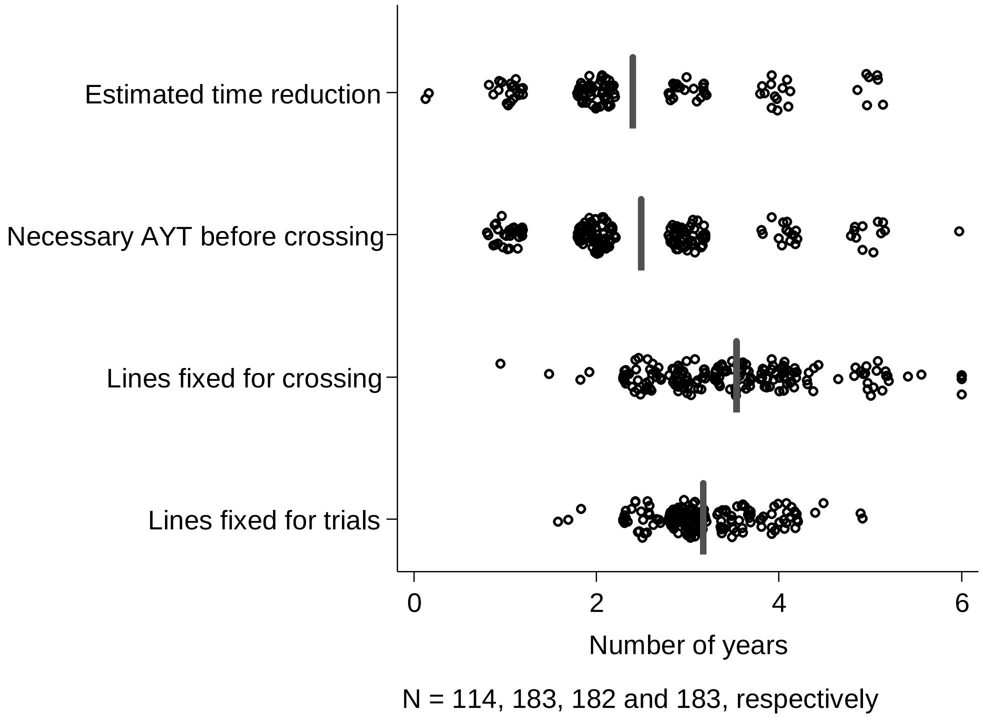

Fig. 2 Perceptions of time regarding breeding. The horizontal lines represent the means. To make comparison possible between different aspects of the breeding process, all concepts are presented here in years taking the median number of seasons per year as 2.

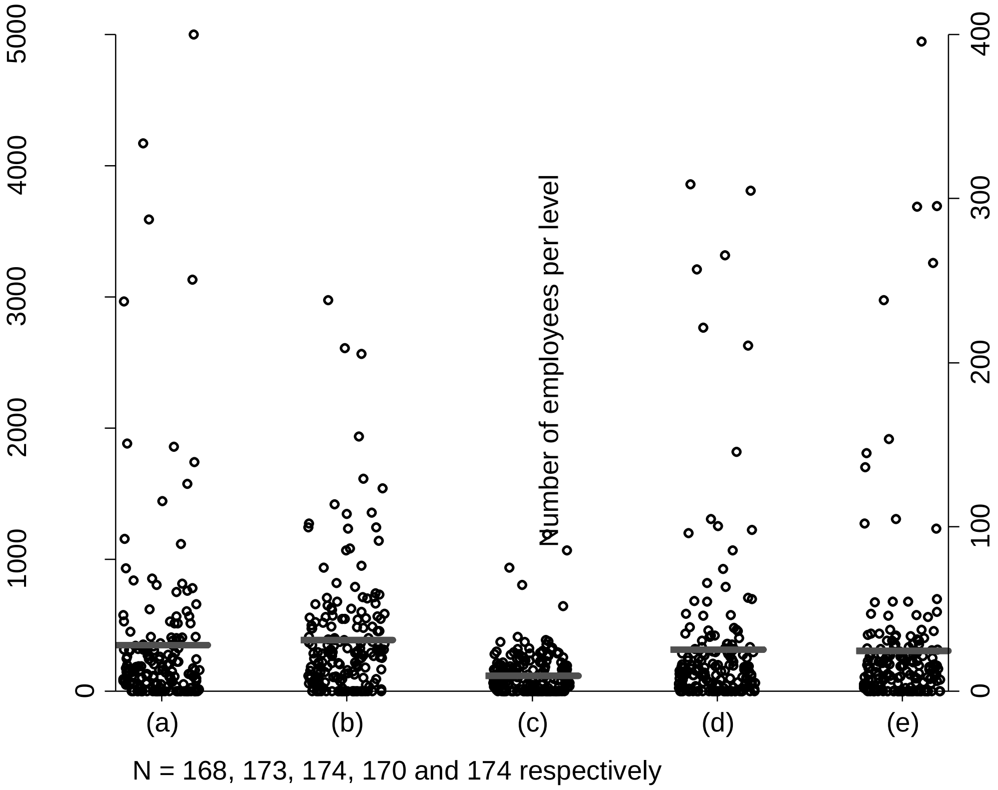

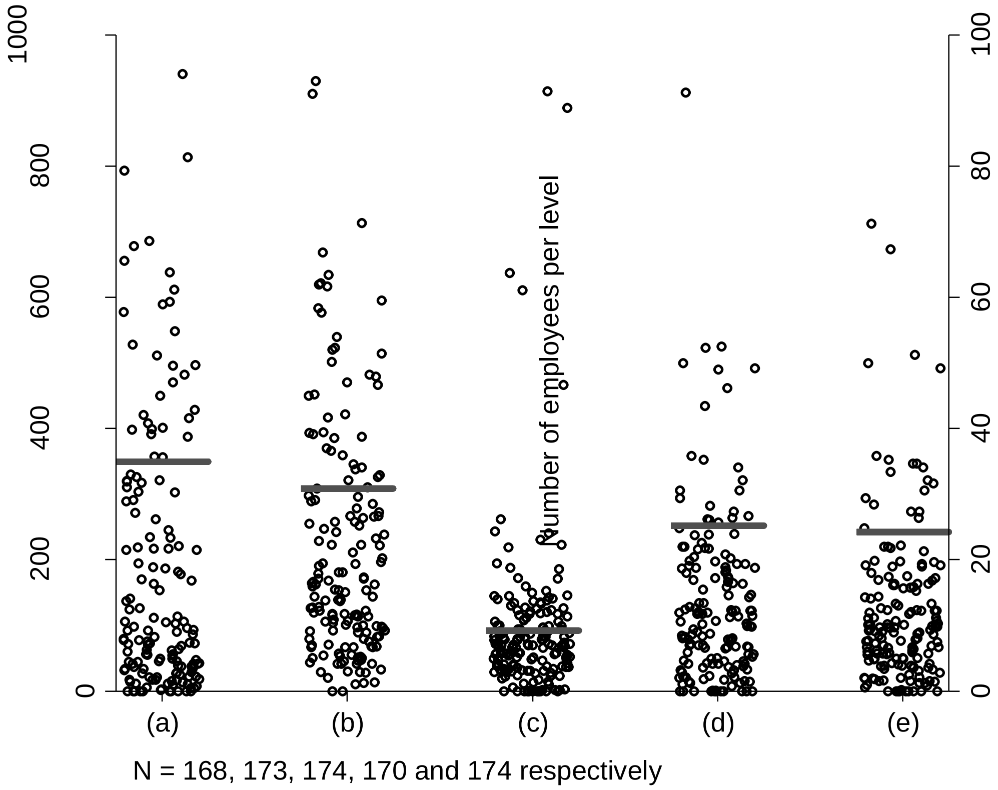

Fig. 3 Staff employment per institute (a), per department (b) and per team (c) and seasonal employment per team for seeding/transplanting (d) and for harvesting (e). Plot (a) is depicted on the left axis while plots (b) to (e) are depicted on the right axis. The horizontal lines represent the means.

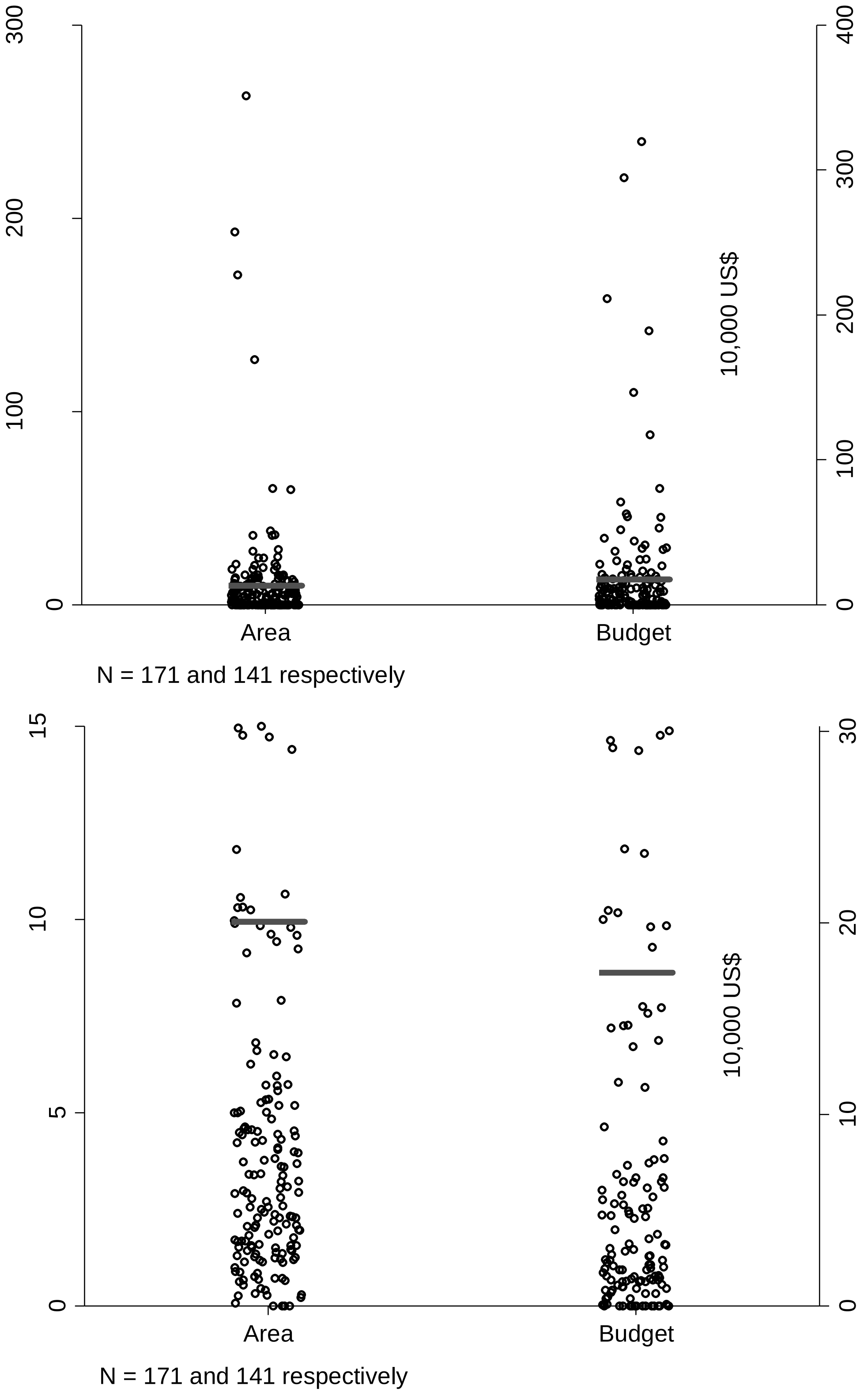

Fig. 4 Boxplot of area of land and budget available to breeders. The horizontal lines represent the means.

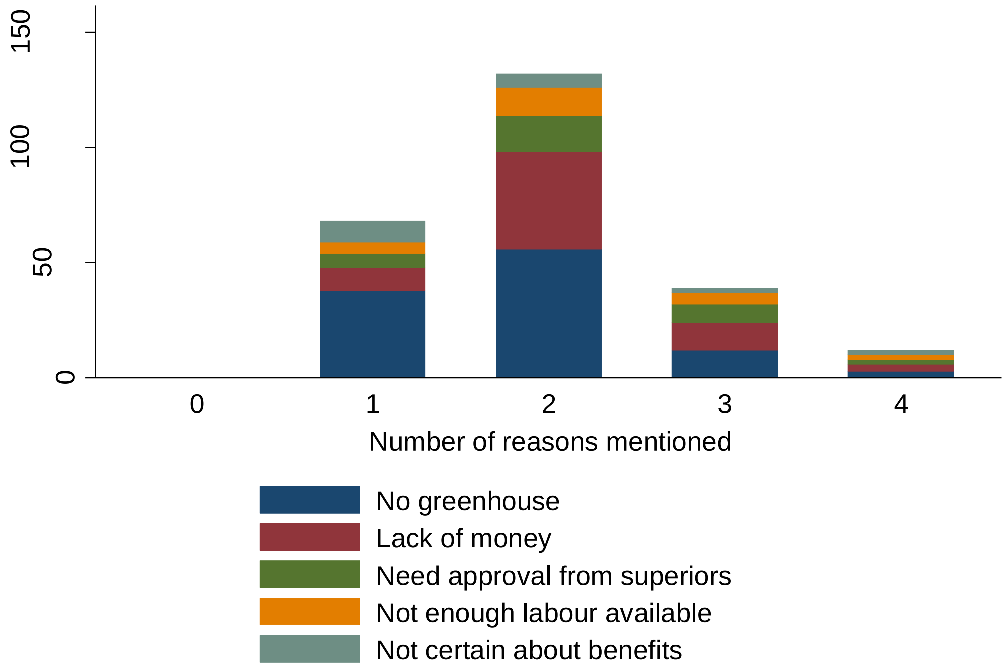

N = 157

Fig. 5 Detailed overview of obstacles specified when willing to adopt RGA.

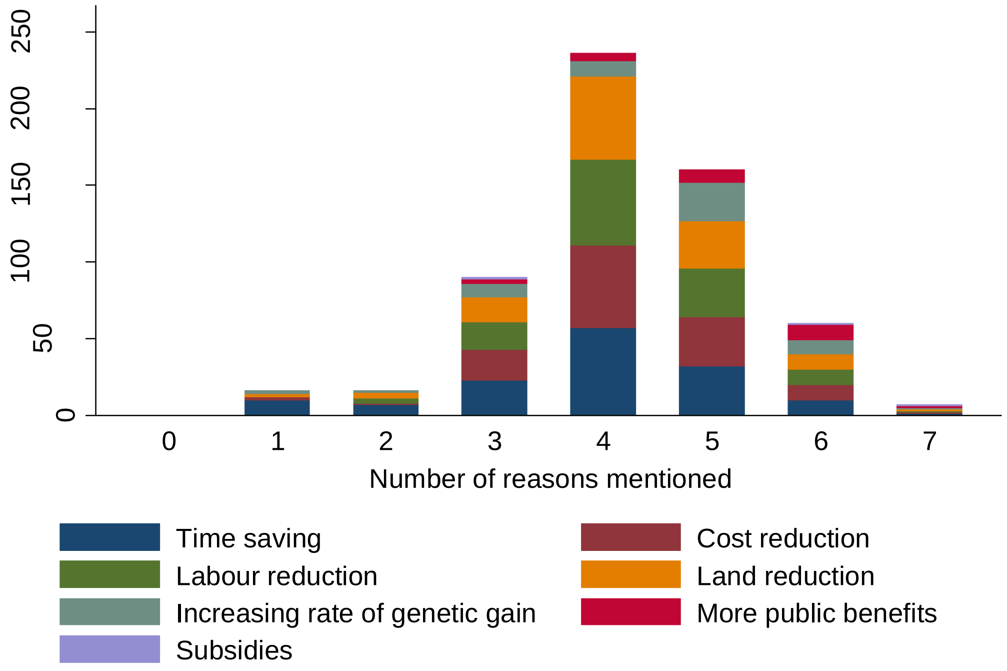

N = 157

Fig. 6 Detailed overview of reasons specified for willingness to adopt RGA.

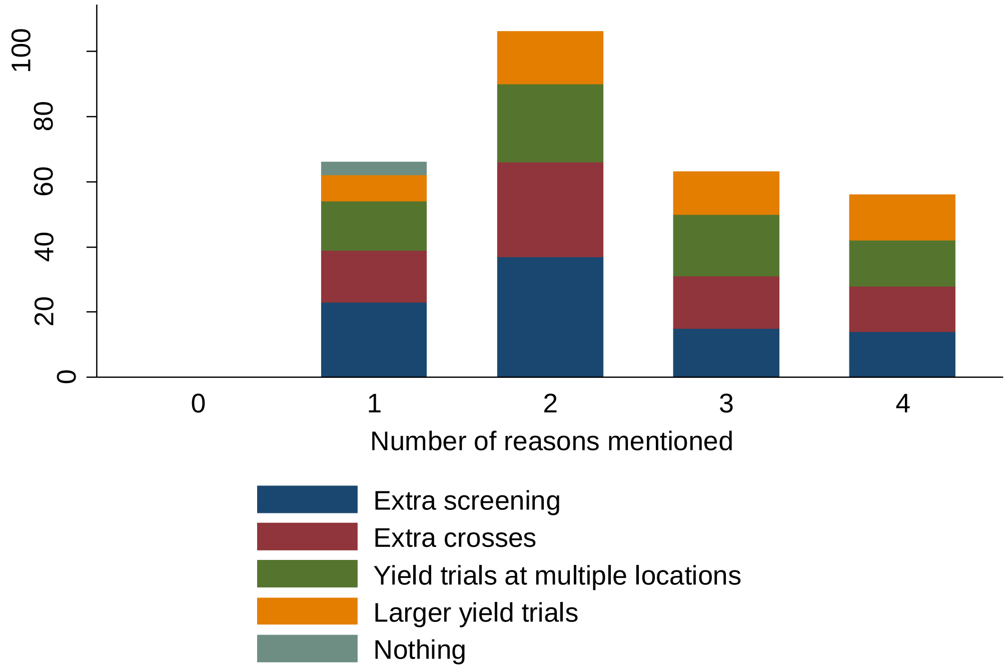

N = 157

Fig. 7 Detailed overview of destination of resource savings specified when willing to adopt RGA.

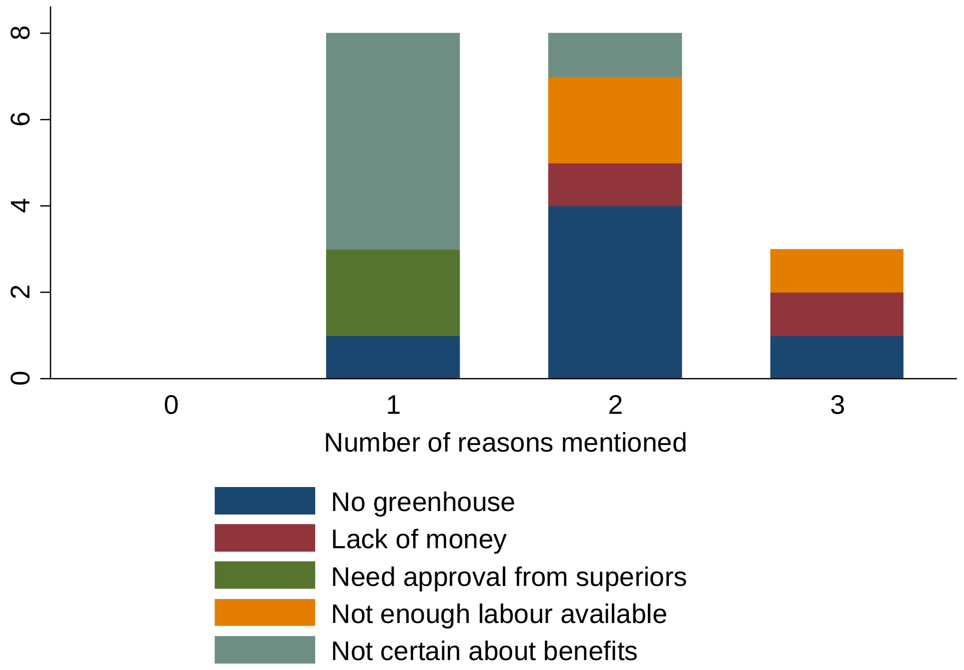

N = 19

Fig. 8 Detailed overview of reasons specified for non-willingness to adopt RGA.

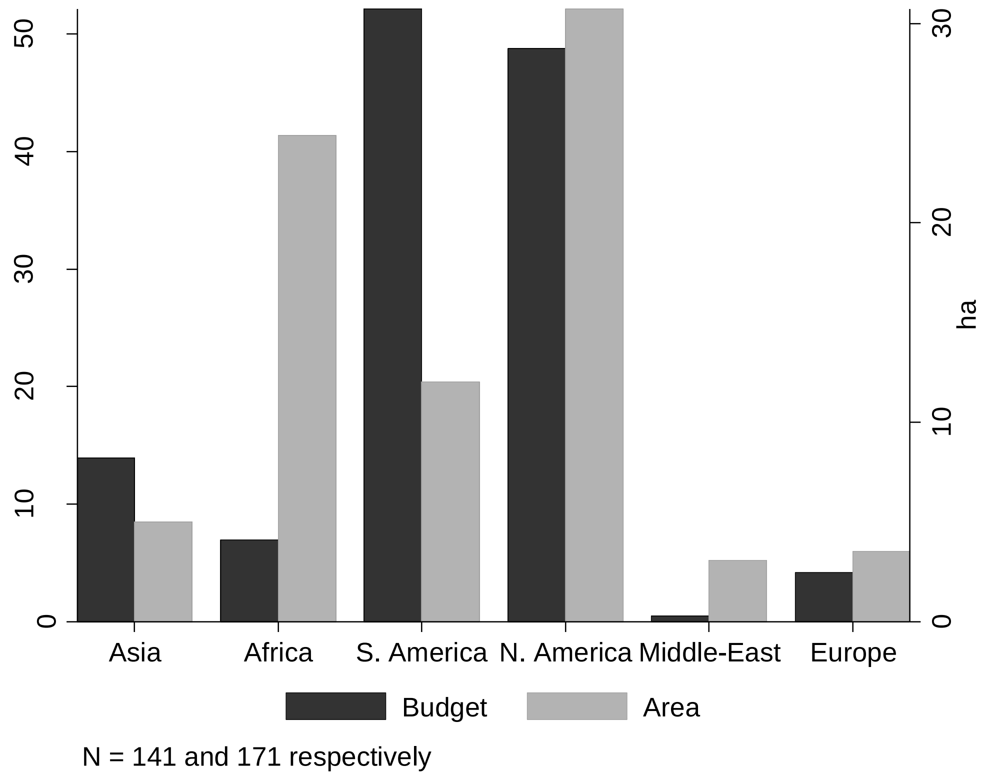

Fig. 9 Mean area of land and budget available to breeders in different continents.

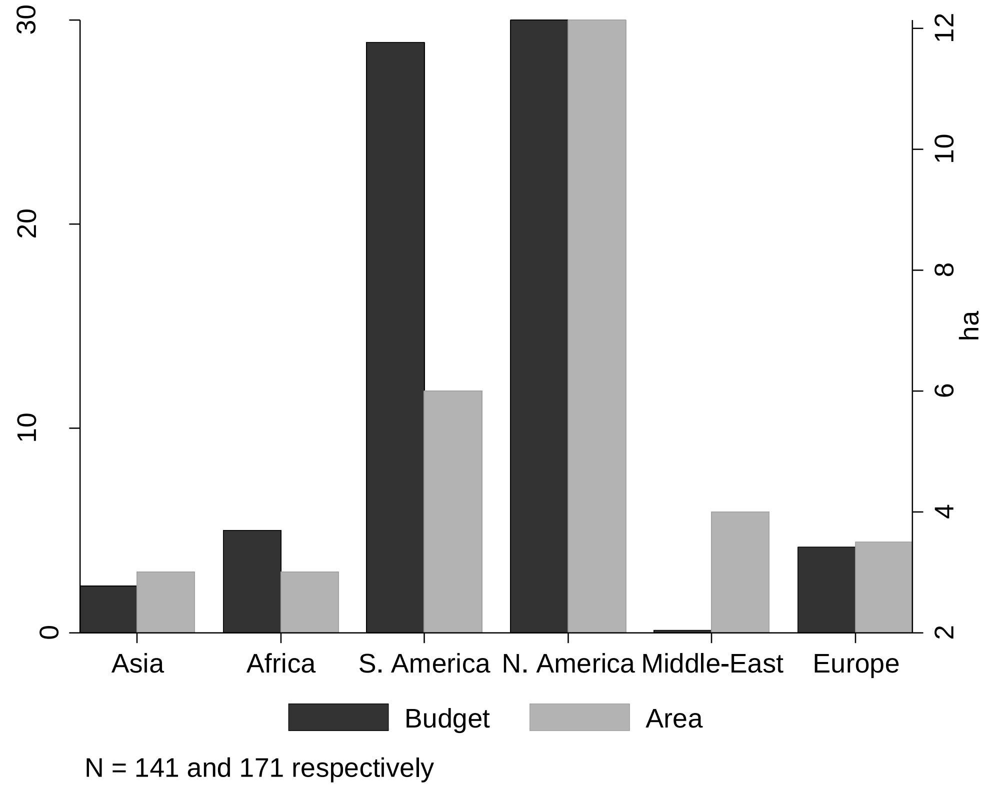

Fig. 10 Median area of land and budget available to breeders in different continents.

Table 1 Country of institute and highest degree obtained by respondents.

	Institute	Highest degree			
Argentina	1	1 (0)	Madagascar	2	-
Australia	-	2 (1)	Malaysia	5	4 (1)
Bangladesh	8	7 (0)	Mali	2	2 (0)
Benin	-	1 (0)	Mexico	1	1 (1)
Bhutan	3	-	Mongolia	1	-
Brazil	5	7 (4)	Nepal	5	3 (1)
Burkina Faso	1	2 (1)	Netherlands	-	4 (2)
Burma	6	5 (0)	Nigeria	1	-
Cameroon	1	1 (0)	Pakistan	12	9 (3)
Chad	1	-	Philippines	8	8 (6)
Chile	1	-	Russia	2	2 (1)
China	3	4 (4)	Rwanda	1	-
Colombia	7	7 (2)	Senegal	4	1 (1)
Congo, Republic of the	1	-	Sierra Leone	1	1 (0)
Cote d'Ivoire	2	1 (0)	South Africa	-	1 (1)
Cuba	-	1 (0)	South Korea	2	3 (1)
Ecuador	1	-	Sri Lanka	1	1 (0)
Egypt	3	4 (4)	Taiwan	-	1 (1)
Ethiopia	2	1 (0)	Suriname	1	-
France	-	1 (1)	Taiwan	1	-
Gambia	1	-	Tanzania	1	2 (1)
Ghana	1	2 (1)	Thailand	15	15 (1)
Guinea-Bissau	1	-	Turkey	2	2 (2)
Guyana	1	-	Uganda	1	-
India	45	51 (45)	United Kingdom	-	4 (2)
Indonesia	5	5 (1)	United States (USA)	7	12 (11)
Iran	3	1 (1)	Uruguay	4	2 (0)
Japan	1	4 (3)	Venezuela	2	1 (0)
Kenya	1	1 (1)	Vietnam	1	-
Laos	1	1 (1)	**Total**	**189**	**189**

Note: Number of PhDs obtained between brackets.
